# The Rhizosphere Microbiome of *Mikania micrantha* Provides Insight Into Adaptation and Invasion

**DOI:** 10.3389/fmicb.2020.01462

**Published:** 2020-07-07

**Authors:** Lijuan Yin, Bo Liu, Hengchao Wang, Yan Zhang, Sen Wang, Fan Jiang, Yuwei Ren, Hangwei Liu, Conghui Liu, Fanghao Wan, Haihong Wang, Wanqiang Qian, Wei Fan

**Affiliations:** ^1^Guangdong Laboratory for Lingnan Modern Agriculture (Shenzhen Branch), Agricultural Genomics Institute at Shenzhen, Chinese Academy of Agricultural Sciences, Shenzhen, China; ^2^Key Laboratory of Protein Function and Regulation in Agricultural Organisms of Guangdong Province, College of Life Science, South China Agricultural University, Guangzhou, China

**Keywords:** Rizosphere bacteria, *Mikania micrantha*, beneficial microbes, nutrition, pathogen

## Abstract

*Mikania micrantha* is a noxious invasive plant causing enormous economic losses and ecological damage. Soil microbiome plays an important role in the invasion process of *M. micrantha*, while little is known about its rhizosphere microbiome composition and function. In this study, we identified the distinct rhizosphere microbial communities of *M. micrantha*, by comparing them with those of two coexisting native plants (*Polygonum chinense* and *Paederia scandens*) and the bulk soils, using metagenomics data from field sampling and pot experiment. As a result, the enrichment of phosphorus-solubilizing bacteria *Pseudomonas* and *Enterobacter* was consistent with the increased soil available phosphorus in *M. micrantha* rhizosphere. Furthermore, the pathogens of *Fusarium oxysporum* and *Ralstonia solanacearum* and pathogenic genes of type III secretion system (T3SS) were observed to be less abundant in *M. micrantha* rhizosphere, which might be attributed to the enrichment of biocontrol bacteria *Catenulispora*, *Pseudomonas*, and *Candidatus Entotheonella* and polyketide synthase (PKS) genes involved in synthesizing antibiotics and polyketides to inhibit pathogens. These findings collectively suggested that the enrichment of microbes involved in nutrient acquisition and pathogen suppression in the rhizosphere of *M. micrantha* largely enhances its adaptation and invasion to various environments.

## Introduction

The rhizosphere is the interface where the complex interactions among soil, microbes, and the host plant are maintained (Philippot et al., 2013). Plants selectively harbor specific microbes through root exudates that contain carbohydrates, amino acids, and organic acid ions, which act as carbon source and nutrients for microbial growth (Reinhold-Hurek et al., 2015). Rhizosphere microbes play pivotal roles in plant growth, nutrient uptakes, and disease suppression (Bulgarelli et al., 2015; Edwards et al., 2015).

Invasive alien species (IAS) could reduce the richness and abundance of native species in the invaded regions, or even dramatically change the local ecological system (Pyšek and Richardson, 2008). Compared with native plants, invasive plants generally performed higher value of inherent traits on physiology, leaf-area allocation, shoot allocation, and growth rate (Van Kleunen et al., 2010) and also exhibit higher abilities to capture and utilize resources at both above- and below-ground processes, such as photosynthesis and nutrition uptake (Van Der Heijden et al., 2008). The soil microbes play important roles in the establishment of invasive plants and might also act as drivers of plant invasions (Dawson and Schrama, 2016). Previous studies showed that invasive plants can recruit different soil microbes to promote their growth over native plants (Reinhart et al., 2003). The indigenous soil microbial communities are altered due to the exotic invading plants (Kourtev et al., 2002). For example, the *Alnus trabeculosa* increased the soil bacterial diversity in the invaded regions (Xueping et al., 2016). Another invasive plant *Centaurea maculosa* enhanced its competitive ability through enriching mycorrhizal fungi that changes soil nutrient availability (Marler et al., 1999). In addition, other studies also showed that many invasive plants have fewer pathogens in rhizosphere than native plants do, escaping from pathogenic agents in soil (Mitchell and Power, 2003). *C. maculosa* could reduce local soil pathogens in invaded regions, therefore investing less in unused defense and more into growth to increase competitiveness against natives (Callaway et al., 2004). An invasive plant may influence soil nutrient content through the soil microbial communities (Piper et al., 2015; Zhao et al., 2019). For example, the invasive tree staghorn sumac changed the structure of soil N2-fixing bacterial communities to enhance soil N availability (Wu et al., 2019). *Solidago gigantea* enhances phosphorus (P) turnover rates in soil (Chapuis-Lardy et al., 2006), and *C. maculosa* increases available P in soil (Thorpe et al., 2006). Invasive plants increased the availability of vital nutrients, thus gaining a competitive advantage, which might be an important contributor to invasion success (Castro-Díez et al., 2014).

*Mikania micrantha* (Asteraceae family), an extremely fast-growing vine, is one of the top 100 worst IAS in the world (Lowe et al., 2000), causing severe substantial damages to natural ecosystems (Day et al., 2016) and economic losses (Macanawai et al., 2012). Several mechanisms have been proposed to explain the success of *M. micrantha* invasion, such as rapid growth caused by high regeneration capacity of each vine node (Li X. et al., 2013) and extraordinary biological characteristics including high seed production and germination (Hu and But, 1994), the strong allelopathic effects on other native plant and soil microbes (Chen et al., 2009), and high nutrient (NPK) turnover rates in soil (Sun et al., 2019; Liu et al., 2020). Recently, we have published the genome of *M. micrantha*, as well as its rhizosphere metagenome, and also found out that the rhizosphere microbes of *M. micrantha* could increase the bioavailable nitrogen content to speed up the nitrogen cycle (Liu et al., 2020), which might contribute to its rapid growth as well as invasion. Enhancing the availability of soil P is also one of the major factors for the success of plant invasion. In recent studies on P acquisition of *M. micrantha*, it was shown that the contents of soil available P and plant tissues of *M. micrantha* were significantly higher than that of native plants. However, very few studies have explained the component and mechanism of P-solubilizing bacteria. We hypothesized that the enrichment of P-solubilizing microorganisms will contribute to the available P in *M. micrantha* rhizosphere. Except for the nutrient acquisition mechanism of plant invasion, the well-known enemy release mechanism that escapes from its natural enemies in its native ranges was also confirmed in other invasive plants. Some invasive plants were not only associated with higher ability of nutrients uptake but also harbored few known pathogens that were more abundant in the rhizosphere of native plants or accumulated pathogens in the soil that are harmful to natives. The research on the invasion mechanism of *M. micrantha* mainly focuses on inherent superiority, allelopathy, and nutrient acquisition, and there is a paucity of research on the influence of pathogenic microorganisms in the *M. micrantha* rhizosphere. We hypothesized that few known pathogens were harbored in *M. micrantha* rhizosphere because of the allelopathy of its leaves and roots. In this study, using these metagenomic data, we investigated the phosphorus solubilizing bacteria and pathogens in the rhizosphere of *M. micrantha*, to better understand the role of the rhizosphere microbiome in *M. micrantha* invasion.

## Materials and Methods

### Experimental Design and Sampling Collection

In order to test the contribution of rhizosphere bacteria to *M. micrantha* invasion, we conducted a pot experiment with *M. micrantha* and its two neighboring native species, namely, *Polygonum chinense* and *Paederia scandens*. These two plants are chosen as the control species because based on the investigation from the field sample, not only are these frequently and stably present in the invasive community of *M. micrantha*, but also the reproduction strategies of these two plants are very similar to those of *M. micrantha* (Sun et al., 2019). The seeds of three plants were germinated and grew to about 10 cm for transplanting. Seedlings of three plants, respectively, planted in the pot (20 cm diameter) filled with natural field soil were collected from the non-invasive area near the invader *M. micrantha* monoculture, which is located in the dry riverbed of Liuxi River, Guangzhou City, Guangdong Province, China. Four treatments (three plants plus a blank control) were replicated six times (two plants per pot with 7 kg fresh soil) and put in a greenhouse.

Three months later, we randomly selected five replicates of each treatment and the rhizosphere soil of three plants and control soil were collected. Plants were removed carefully and shaken lightly; then, the soil remaining attached to the root surface was collected with sterile water. The separated soil solution was centrifuged at 8000 r/min for 10 min to collect soil samples. The collected soils were stored at −80°C until use for microbial community analysis. *M. micrantha* is an ecologically harmful weed in the natural environment. We chose the natural field of *M. micrantha* monoculture with the dominant two coexisting native plants (*P. chinense* and *P. scandens*) in the dry riverbed of Liuxi River in Guangzhou City. We separated five (5 m by 5 m) sampling plots by more than 200 m and used the same method of pot experiment to collect 15 rhizosphere samples of three plants and five control samples, which is in the uninvaded area near *M. micrantha* monoculture by more than 500 m, for a total of 20 samples.

### DNA Extraction and Sequencing

A combination of bacteria cell lysis steps was applied before DNA extraction. The soil microbial cells were subjected to six freeze–thaw cycles (alternating vortex for 5 min, then liquid nitrogen for 5 min, and incubation at 65°C for 5 min). Next, DNA was extracted from all samples using the PowerSoil DNA isolation kit following the manufacturer’s protocol (MO BIO Laboratories, QIAGEN Inc., United States). The DNA quality and quantity were checked by the Nanodrop and Qubit device, and the DNA quantity of each sample was at least 1 μg. Then, DNA fragments (200–400 bp) were processed by ultrasonic instrument (Thermo Fisher Scientific, Covaris S220). The library was constructed using TruSeq DNA PCR-Free Library Prep Kit as per standard protocol (Illumina, United States) and then sequencing was performed on Illumina HiSeq 2500 with each sample having about 10 Gb sequencing data.

### Metagenomic Analyses

The raw reads were cleaned by removing adaptor sequences, low-quality sequences, host sequences, and unpaired reads by in-house software clean_adapter, clean_lowqual, and filter_unpaired_reads.pl^1^, resulting in a clean and high-quality reads data with average error rate < 0.001. Then, the clean reads from each sample and pooled for four groups (*M. micrantha*, *P. chinense*, *P. scandens*, and control) were assembled by Megahit (v1.1.3). After filtering the contig length less than 500 bp, gene prediction was performed using MetaProdigal (v2.6.3), and then we filtered out the gene models with cds length less than 102 bp. The protein models of each sample and each group were also performed using MetaProdigal (v2.6.3). The non-redundant gene catalog was obtained using the genes predicted from each sample and each group by cd-hit-est (v4.6.6) with the criteria of identity > 95%, and overlap > 90% (parameter “−c 0.95 −n 10 −G 0 −aS 0.9”). The non-redundant protein catalog was obtained from the combination of protein files of each sample and each group by in-house software fishInWinter.pl^2^.

To generate the taxonomic information, the non-redundant protein sequences were aligned against the NCBI-NR database using DIAMOND (v0.8.28.90) software with the parameter “blastp –evalue 10 –max-target-seqs 250” (Buchfink et al., 2015). CARMA3 software (parameter “carma –classify-blast –type p –database p”) was used to assign the taxonomic annotation of the unigenes (Gerlach and Stoye, 2011). Thus, we obtained the non-redundant genes and their corresponding species classification. To obtain functional information for the gene set, the non-redundant protein sequences were searched (*E* value < 1e-5) against the KEGG protein database (release 79) using DIAMOND software (Kanehisa et al., 2004). To calculate the relative gene abundance, the clean reads from each sample were aligned against the non-redundant gene catalog by BWA-MEM (alignment length ≥ 50 bp and identity > 95%) (Li and Durbin, 2009). The alignments were parsed to produce the reads count abundance (Huang et al., 2018). Based on the taxonomic assignments using CARMA3, the relative abundance of each phylum, genus, species, and KO was calculated by summing the abundances of corresponding genes belonging to each category per sample. Similarly, the relative abundance profile of genes was also summarized into KEGG functional profiles for the functional analysis.

### Functional Bacteria and Genes Collection

The bacteria and genes involved in soil microbial P-solubilizing and mineralization, pathogen, and defense were searched based on previous publications and are shown in Supplementary Tables S1–S6 (Weller et al., 2002; Garbeva et al., 2004; Beth Mudgett, 2005; Raaijmakers and Mazzola, 2012; Sharma et al., 2013; Raj et al., 2014; Alori et al., 2017; Han et al., 2018; Dai et al., 2019). The names, KOs, and functions of the genes associated with P solubilizing and mineralization, type III secretion/effector systems, and polyketide synthase (PKS) are shown in Supplementary Tables S2, S4, S6, respectively.

### Microbial Composition Analysis

At the gene level, Shannon index was used to analyze microbial alpha diversity using the non-redundant genes of individual samples. The overall differences in the bacterial community structures were calculated by non-metric multidimensional scaling (NMDS) using non-redundant genes of individual samples based on Bray–Curtis dissimilarity values and implemented in in R package “Phyloseq.”

### Statistical Analysis

Based on the relative abundance profiles at the phyla or genera level, the significantly differential abundances in the control soil and rhizospheres of *M. micrantha*, *P. chinense*, and *P. scandens* were determined using Kruskal–Wallis test with Dunn’s multiple comparison (BH methods for multiple tests adjustment). The relative abundance of microbial species and functional genes involved in P solubilization, pathogens, and defense in the control soil and rhizospheres of three plants is compared using Kruskal–Wallis test with Dunn’s multiple comparison (BH methods for multiple tests adjustment).

## Results

### Microbial Structure of the Rhizosphere Microbiome

**FIGURE 1:**
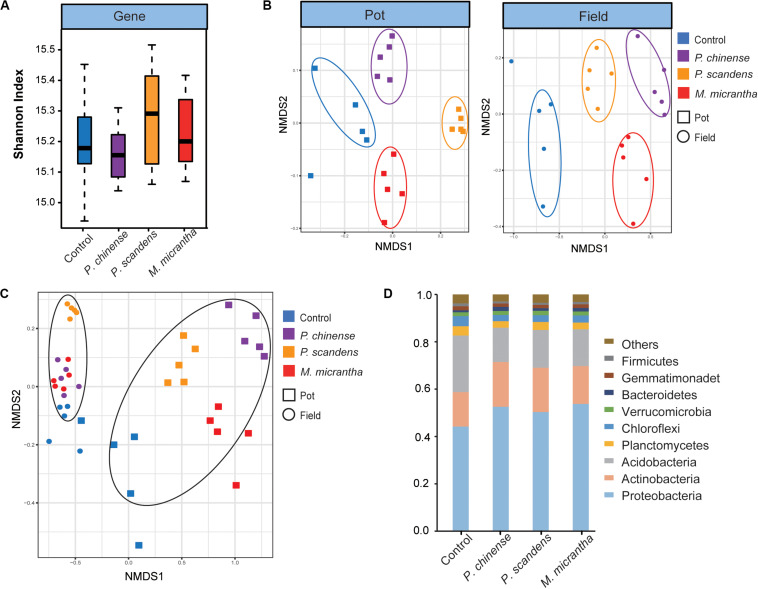
Microbial structure in the rhizosphere of *Mikania micrantha* (*M. micrantha*), *Polygonum chinense* (*P. chinense*), and *Paederia scandens* (*P. scandens*), and control (bulk) soil. **(A)** Comparison of the microbial diversity at the gene level. **(B)** The non-metric multidimensional scaling (NMDS) plot of microbial communities at both pot experiment and invaded site. The analysis was based on Bray–Curtis dissimilarities at the gene level. **(C)** The NMDS plot of microbial communities in all samples, based on Bray–Curtis dissimilarities at the gene level. **(D)** Relative abundances at the phylum level.

Using the metagenomic data and non-redundant gene set of *M. micrantha* genome project, deposited in NCBI (SRR8936416–SRR8936475) and AGIS ftp-site^3^, we investigated the microbial structure of the rhizosphere of *M. micrantha* and two native plants (*P. chinense* and *P. scandens*). The microbial alpha diversity (Shannon index) at the gene level showed no significant difference between the control soil (bulk soil) and rhizospheres (*P* > 0.05) (Figure 1A). However, at the gene level, the NMDS analysis revealed the distinct microbial community differences among the rhizospheres of *M. micrantha*, two native plants, and control soil at both pot experiment and invaded site (Figure 1B). Moreover, the NMDS plots showed that there was a clear separation between the pot experiment and field invaded site, indicating that rhizosphere microbial community was largely influenced by environmental conditions (Figure 1C). The dominant prokaryotic phyla found in the control and rhizosphere community included *Proteobacteria*, *Actinobacteria*, *Acidobacteria*, *Planctomycetes*, and *Chloroflexi* (Figure 1D), which was consistent with previous studies (Lu-Irving et al., 2019). The community differences between the control soil and rhizospheres of *M. micrantha* and native plants were also explored. *Proteobacteria* and *Actinobacteria* occupied higher percentages than in the control soil, whereas *Acidobacteria* has lower percentages (*P* < 0.05, Dunn test) in the rhizospheres (Figure 1D). This suggests that some bacteria from bulk soil are selected to inhabit in the rhizospheres.

### Distinctive Enrichment of Plant Microbes

**FIGURE 2:**
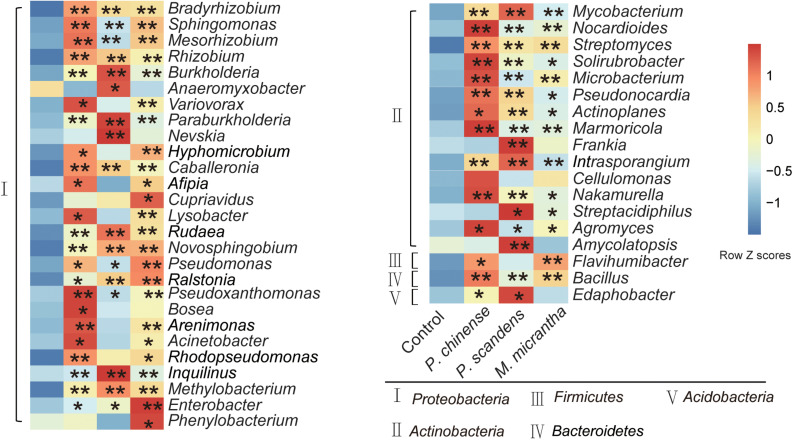
Comparison of average relative abundance at the genus level in rhizospheres of *M. micrantha* and two native plants. The relative abundance of each genus was colored according to the row *z* score ((value - row mean)/row standard deviation). The comparisons of microbes in plant rhizospheres and control soil were compared by the Kruskal–Wallis test with Dunn’s multiple comparison test (*P < 0.05 and **P < 0.01).

The microbial compositions of the rhizosphere of *M. micrantha* and the two native plants were analyzed at the genus level, at both pot experiment and invaded site. From the metagenomic data, genes could be classified to the genus level by CARMA3 software. The relative abundance of genus in each sample was calculated according to reads count at the genus level. In total, the top 69 genera (relative abundance > 0.01%) accounted for 94.8% of the total relative abundance of classified genera, and 45 of them were enriched (*P* < 0.05, Dunn test) in rhizospheres compared to the bulk soil, most of which belong to *Proteobacteria* and *Actinobacteria* (Figure 2). Moreover, 30 genera were all enriched in rhizospheres of three plants, including *Bradyrhizobium*, *Mesorhizobium*, *Rhizobium*, *Burkholderia*, *Paraburkholderia*, *Methylobacterium*, *Novosphingobium*, *Pseudomonas*, *Enterobacter*, *Bacillus*, *Nocardioides*, and *Streptomyces*, many species of which were known as plant beneficial microbes that can facilitate nutrition acquisition, improve resistance to abiotic stress, and control phytopathogens (Figure 2) (Ahemad and Kibret, 2014; Cordovez et al., 2018; Vives-Peris et al., 2018). The enrichment of these plant microbes might facilitate plant assembling beneficial endosphere bacteria from the rhizosphere soil.

Previous studies have shown that the plant species and varieties could influence the composition of their rhizosphere (Philippot et al., 2013; Zhang et al., 2019). In our study, the microbe enrichment in rhizospheres of *M. micrantha* and native plants was also distinctive. *Enterobacter*, *Pseudomonas*, *Cupriavidus*, and *Phenylobacterium* relatively occupied higher percentages in *M. micrantha* rhizosphere compared to *P. chinense* and *P. scandens* rhizospheres (Figure 2). Many species belonging to *Enterobacter* and *Pseudomonas* (Meena et al., 2017; Zheng et al., 2019) are well known plant-beneficial microbes, and *Cupriavidus* and *Phenylobacterium* species were reported to participate in the mineralization of soil organic P and degrade organic material (De La Cruz-Barrón et al., 2017). In comparison, *P. chinense* rhizosphere was enriched with *Variovorax*, *Bosea*, and *Acinetobacter*, and some species of which could inhibit pathogens and supply N for plant growth (Rilling et al., 2018; Bruisson et al., 2019), and *P. scandens* rhizosphere was enriched with *Anaeromyxobacter*, *Frankia*, *Streptacidiphilus*, and *Amycolatopsis* (Figure 2), with nitrogen-fixing (Chaia et al., 2010) and antimicrobial activity (Buszewski et al., 2018). In summary, although many bacteria are shared among three plant species, each plant still recruits distinctive microbes in rhizosphere, possibly due to their different root exudates.

### Enrichment of *Pseudomonas* and *Enterobacter* to Enhanced Phosphorus Solubilization

Phosphorus (P), is an essential element for plant growth and development (Sharma et al., 2013), playing important roles in many metabolic processes of plant, including photosynthesis, signal transduction, energy transfer, respiration, macromolecular biosynthesis (Khan et al., 2010), and nitrogen fixation (Kouas et al., 2005). Microorganisms are major members of the soil P cycle, improving available P to plants (Khan et al., 2009). We have previously reported that the available P content in *M. micrantha* rhizosphere was significantly higher than that in the rhizosphere of two native plants (Liu et al., 2020).

**FIGURE 3:**
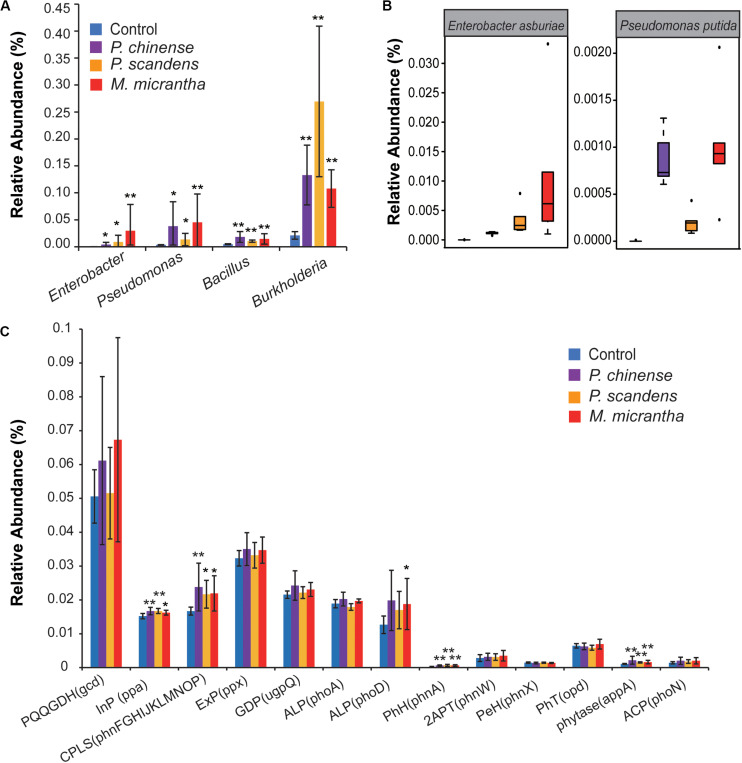
Enhanced soil-borne available P in the rhizosphere of *M. micrantha* and two native plants. **(A)** The relative abundance of phosphate bacteria. **(B)** The relative abundance of *Enterobacter asburiae* and *Pseudomonas putida*. On each boxplot, the central mark indicates the median, the bottom and top edges of the box indicate the interquartile range (IQR), and the whiskers represent the maximum and minimum data points. **(C)** The relative abundance of genes coding for P solubilization and P mineralization. PQQGDH, quinoprotein glucose dehydrogenase; InP, inorganic pyrophosphatase; CPLS, C-P lyase subunit; ExP, exopolyphosphatase; GDP, glycerophosphoryl diester phosphodiesterase; ALP, alkaline phosphatase; PhH, phosphonoacetate hydrolase; 2APT, 2-aminoethylphosphonate-pyruvate transaminase; PeH, phosphonoacetaldehyde hydrolase; PhT, phosphotriesterase; ACP, acid phosphatase. The C-P lyase subunit was calculated as the total abundances of gene *phnF*, *phnG*, *phnH*, *phnI*, *phnJ*, *phnK*, *phnL*, *phnM*, *phnN*, *phnO*, and *phnP*. Error bars indicate average value ± SEM of indicated replicates. The pairwise comparisons of rhizosphere in each plant and control soil were used by the Kruskal–Wallis test with Dunn’s multiple comparison test (*P < 0.05 and **P < 0.01).

Phosphorus solubilizing microorganisms (PSMs), such as *Pseudomonas*, *Bacillus*, *Enterobacter*, and *Burkholderia* (Babalola and Glick, 2012), can increase soil available P via solubilization and mineralization of unavailable P in organic matter and minerals. These PSMs were enriched in the rhizospheres of *M. micrantha*, *P. chinense*, and *P. scandens* (Figure 3A); however, the relative abundance of PSMs is different. *Enterobacter* was most highly enriched in *M. micrantha* rhizosphere, with its average relative abundance 7-fold and 100-fold higher than that in *P. chinense* and *P. scandens* rhizosphere, respectively (Figure 3A). Similarly, the average relative abundance of *Pseudomonas* was also 1.5-fold and 13-fold higher than those in *P. chinense* and *P. scandens* rhizosphere, respectively (Figure 3A). In the invaded field site, the plant-growth promotion bacteria *Pseudomonas putida* (Możejko-Ciesielska and Serafim, 2019) and *Enterobacter asburiae* (Teng et al., 2019) were more abundant in *M. micrantha* rhizosphere (Figure 3B). On the other hand, *Bacillus* and *Burkholderia* were more enriched in rhizosphere of *P. chinense* and *P. scandens* (1.2- and 2.5-fold that in *M. micrantha* rhizosphere), which might also contribute to the solubilization of soil unavailable phosphorus. Taken together, the recruitment of these PSM would help to increase the available P content in *M. micrantha* rhizosphere.

Next, the genes generally contained in PSM were analyzed, including those coding for P mineralizing and solubilizing enzymes (Dai et al., 2019). The genes coding for organic P mineralization, such as C-P lyase, phosphonoacetate hydrolase, and phytase, as well as the genes coding for inorganic pyrophosphatase responsible for the inorganic P solubilization, were all enriched in rhizosphere of *M. micrantha* and two native plants (*P* < 0.05) (Figure 3C). The genes involved in alkaline phosphatase phoD were more abundant in rhizosphere of *M. micrantha* (*P* = 0.045) and *P. chinense* (*P* = 0.07), whereas phoA showed no significant difference (*P* > 0.05) (Figure 3C). The relative abundance of other genes showed no significant difference (*P* > 0.05) (Figure 3C). These results indicated that the rhizosphere microbes in *M. micrantha* and *P. chinense* may contribute to available P through the similar P mineralization mechanism in terms of alkaline phosphatase.

### Fewer Pathogens in *M. micrantha* Rhizosphere Microbiota

The plant-associated microbiome, as the second genome of the plant, has great influence on plant growth and health (Berendsen et al., 2012). To suppress the pathogen attack, plants could be able to recruit protective microorganisms in the rhizosphere, as the complement of the plant innate immune system (Mendes et al., 2014).

**FIGURE 4:**
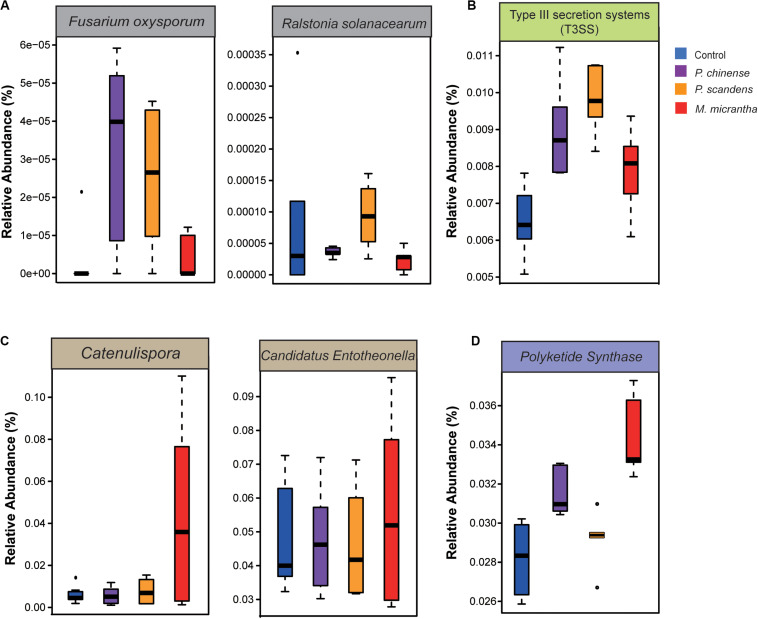
Fewer pathogens and more biocontrol bacteria in *M. micrantha* rhizosphere. **(A)** The relative abundance of pathogens of *Fusarium oxysporum* and *Ralstonia solanacearum*. **(B)** The relative abundance of pathogenic genes of type III secretion systems (T3SS). **(C)** The relative abundance of biocontrol bacteria of *Catenulispora* and *Candidatus Entotheonella*. **(D)** The relative abundance of genes coding for type II polyketide synthase (PKS). On each boxplot, the central line indicates the median, the bottom and top edges of the box indicate the interquartile range (IQR), and the whiskers represent the maximum and minimum data points.

The aggressive soil-borne pathogens were analyzed (Supplementary Table S3). Although many pathogens could not be detected in our data, we found that the pathogens of *Fusarium oxysporum* (Srinivas et al., 2019) and *Ralstonia solanacearum* (Genin and Denny, 2012) were enriched in *P. chinense* (sevenfold and twofold) and *P. scandens* (sixfold and fourfold) rhizosphere compared to *M. micrantha* rhizosphere (Figure 4A). Besides, the genes involved in the host–pathogen interactions (Supplementary Table S4) [type III secretion system (T3SS)] were more abundant in the rhizosphere of *P. scandens* (*P* = 0.04) than in *M. micrantha* rhizosphere (Figure 4B). Plants could inhibit pathogen attack by secreting antimicrobials or recruiting the biocontrol microbes that have the relevant antimicrobial gene cluster (Berendsen et al., 2012). The biocontrol microbes (Supplementary Table S5), such as *Pseudomonas*, *Catenulispora*, and *Candidatus Entotheonella*, were more abundant in rhizosphere of *M. micrantha* than that in two native plants (Figures 3A, 4C). It is known that some species belonging to *Catenulispora*, *Pseudomonas*, and *Candidatus Entotheonella* could suppress pathogen by producing antibiotics and polyketides (Zettler et al., 2014; Kurnia et al., 2017). In our results, type II PKS genes (Supplementary Table S6) that were involved in synthesizing aromatic polyketides that could control plant disease (Han et al., 2018) were also enriched in *M. micrantha* rhizosphere (*P* = 0.002) (Figure 4D), whereas type III PKS genes were not different among three plants. These results indicated that the biocontrol bacteria might contribute to the less pathogens by antimicrobial aromatic polyketides in *M. micrantha* rhizosphere.

## Discussion

The success of plant invasion depends on enemy release, enhanced nutrient acquisition, and adaptations to the physical environment (Dawkins and Esiobu, 2016). Recently, increased attention has been paid to the interactions between soil microbes and plant invasions (Dawkins and Esiobu, 2018). In this study, we investigated the role of soil microbes in plant invasions by comparing the taxonomic and functional difference of rhizosphere community between the invasive plant *M. micrantha* and two native plants (*P. chinense* and *P. scandens*) at invaded field site and pot experiment. Since the pot experiment lasted only 3 months, and each plant grew independently without competition, obvious microbial differences between pot experiment and invaded site were observed (Figure 1C). However, many plant-associated microbes were enriched in rhizospheres both in the pot experiment and the invaded site, and these genera were generally higher in the invaded field than those in the pot experiment, indicating their important roles in the natural environment. The interactions between an invasive plant and associated soil communities changed across the invaded range (Nunes et al., 2019). In our study, we found that there is a difference of *M. micrantha* rhizosphere between the pot experiment and the field site. As a plant killer, more field samples of the rhizosphere microbes of *M. micrantha* across latitudinal gradients in its invaded range should be analyzed to understand the interactions between its performance and soil microbes. This could provide an important basis for controlling its spread. By comparing the microbes in the rhizospheres and in the control soil, we found that the relative abundance of *Proteobacteria* and *Actinobacteria* was higher in the rhizosphere than in the control soil, whereas *Acidobacteria* was more abundant in control soil (Figure 1C). The distinct enrichment may be attributed to the abundant nutrients in rhizosphere, which promote the copiotrophic microorganisms (Ling et al., 2017) and the inhibited growth of oligotrophic microorganisms (Fierer et al., 2007).

The competition of invasive species with native species depends largely on the abilities of accession in resources (Seabloom et al., 2003). P is an essential macronutrient for plant growth and development (Lidbury et al., 2016), and microorganisms play an important role in soil P cycling and in regulating P availability (Dai et al., 2019). In this study, we found that *Enterobacter* and *Pseudomonas* might contribute to the increased soil available P content, and helped *M. micrantha* to outcompete native species and ultimately facilitate plant invasion (Figure 3A). Although the gene of *gcd* was not significantly different when the field and potted samples were analyzed together (Figure 3C), it was found significantly enriched in rhizosphere of *M. micrantha* (*P* = 0.0008) and *P. chinense* (*P* = 0.02) (Supplementary Figure S1) in the invaded site. The relative abundance of the *gcd* gene in *M. micrantha* rhizosphere was 1.2-fold of that in *P. chinense* rhizosphere and 1.5-fold of that in *P. scandens* rhizosphere in the field. Besides, even genes coding for alkaline phosphatase were at a similar level in the rhizosphere of *M. micrantha* and *P. chinense*, and the highly elongated, deep, and extensive root system of *M. micrantha* may still promote the efficient uptake of the released available P in soil.

Invasive plants may benefit from introduction to new regions where they can escape pathogens on the native ranges (Lu-Irving et al., 2017). Recently, Ramirez et al. (2019) found that the range-expanding plants harbored fewer pathogens compared to native species in the new range, through the analysis of the microbiome of European continental range-expanding plant species along a latitudinal gradient. This result was consistent with our study, which revealed that the pathogens and pathogenic genes, including the soil-borne pathogen *F. oxysporum* and *R. solanacearum*, as well as T3SS, were depleted in *M. micrantha* rhizosphere compared to the native plants (Figures 4A,B). Correspondingly, many biocontrol bacteria such as *Catenulispora*, *Pseudomonas*, and *Candidatus Entotheonella*, which release antibiotics and polyketides to inhibit pathogens (Kurnia et al., 2017; Mori et al., 2018), were enriched in *M. micrantha* rhizosphere. In addition, Mikania sesquiterpene lactones (STLs) have allelopathic effects on native plants and antibacterial activities (Li Y. et al., 2013), which may also contribute to the fewer pathogens in *M. micrantha* rhizosphere. In summary, the fewer pathogens and more protective microorganisms that inhabit the *M. micrantha* rhizosphere potentially benefit root growth and nutrient uptake, thus possibly enabling the successful invasion. However, there is a lack of difference in the soil microbes in *M. micrantha* between the origin and invaded one. Evidences for the resource availability and pathogen release in soil of invasive plants would require combined tests in the native and invaded ranges. Hence, in order to comprehensively understand the role of soil microorganisms in *M. micrantha* invasion, the metagenome of *M. micrantha* rhizosphere in the native range and the differences to their introduced range would need to be studied in the future. Although we showed the differences of microbial community and functional genes among the rhizosphere of three plants, the observed changes would require further experimental study.

## Conclusion

*Mikania micrantha* rhizosphere has a distinct bacteria community structure that is clearly separated from the native plants and the bulk soil. Although some common microbes are observed in the rhizosphere of both *M. micrantha* and two native plants, *M. micrantha* rhizosphere specifically recruited *Cupriavidus*, *Enterobacter*, *Pseudomonas*, and *Phenylobacterium*, which played important roles in resource acquisition, plant hormone regulation, and pathogen suppression. On the other hand, the rhizosphere of native plants *P. chinense* and *P. scandens* recruited some other distinctive plant microbes. According to our analysis, the previously found higher soil available P content in *M. micrantha* rhizosphere was possibly contributed by the enrichment of P-solubilizing bacteria *Enterobacter* and *Pseudomonas*. Moreover, pathogens including *F. oxysporum* and *R. solanacearum* and pathogenic genes of T3SS were less abundant in *M. micrantha* rhizosphere compared to the two native plants. In contrast, the biocontrol bacteria such as *Catenulispora*, *Pseudomonas*, and *Candidatus Entotheonella*, as well as the PKS genes were enriched in *M. micrantha* rhizosphere to develop antibacterial activities. Taken together, these findings deepen our understanding of the microbial composition and function in *M. micrantha* rhizosphere, as well as the two native plants, and thus provide useful information that would help develop efficient technologies to control the invasion of *M. micrantha*.

## Data Availability Statement

Publicly available datasets were analyzed in this study. These data can be found in the NCBI under the accession numbers SRR8936416–SRR8936475.

## Author Contributions

BL, WF, and WQ conceived the study. LY, HeW, YZ, FJ, and SW collected the samples and analyzed the data. YR, CL, HL, WQ, HaW, and FW provided suggestions and helped in the checking. YZ, SW, BL, WQ, and WF helped to revise the manuscript. All authors contributed to the article and approved the submitted version.

## Conflict of Interest

The authors declare that the research was conducted in the absence of any commercial or financial relationships that could be construed as a potential conflict of interest.

## Footnotes

1 https://github.com/fanagislab/DBG_assembly/tree/master/clean_illumina

2 https://github.com/fanagislab/bioinfo_versatiles/blob/master/fishInWinter.pl

3 ftp://ftp.agis.org.cn/Mikania_micrantha/
