# Supplementary material for: The Rhizosphere Microbiome of Mikania micrantha Provides Insight Into Adaptation and Invasion
Source: Front Microbiol. 2020 Jul 7;11:1462. doi: 10.3389/fmicb.2020.01462 (PMC7359623; doi:10.3389/fmicb.2020.01462)
Supplement: Supplementary file 1 [file Table_1.DOCX]

**Table S1.** The average relative abundance of phosphorus solubilizing microorganisms (PSM) in rhizosphere of *M. micrantha* and two native plants.

|  | Bacteria | Control | *P. chinense* | *P. scandens* | *M. micrantha* |
| --- | --- | --- | --- | --- | --- |
| Phosphorus solubilizing microorganisms (PSM) | *Azotobacter* | 1.42E-06 | 2.00E-06 | 1.53E-06 | 2.00E-06 |
|  | *Bacillus* | 4.88E-05 | 1.83E-04 | 1.04E-04 | 1.47E-04 |
|  | *Burkholderia* | 2.11E-04 | 1.33E-03 | 2.70E-03 | 1.08E-03 |
|  | *Enterobacter* | 1.02E-06 | 4.06E-05 | 9.06E-05 | 2.97E-04 |
|  | *Erwinia* | 9.50E-08 | 5.87E-07 | 4.59E-07 | 1.04E-06 |
|  | *Paenibacillus* | 6.30E-05 | 4.91E-05 | 5.16E-05 | 5.04E-05 |
|  | *Pseudomonas* | 3.45E-05 | 3.82E-04 | 1.34E-04 | 4.53E-04 |
|  | *Rhodococcus* | 2.97E-05 | 4.21E-05 | 4.01E-05 | 3.24E-05 |
|  | *Salmonella* | 0.00E+00 | 7.83E-07 | 5.82E-07 | 1.68E-06 |
|  | *Serratia* | 2.60E-07 | 8.82E-07 | 7.73E-07 | 1.36E-06 |
|  | *Thiobacillus* | 3.23E-06 | 2.73E-06 | 2.89E-06 | 3.38E-06 |

**Table S2.** The function descriptions and KO number of the solubilizing P genes referring to KEGG database.

| genes | KO | Description |
| --- | --- | --- |
| *gcd* | K00117 | quinoprotein glucose dehydrogenase |
| *phnN* | K05774 | C-P lyase subunit, ribose 1,5-bisphosphokinase |
| *phnL* | K05780 | C-P lyase subunit, alpha-D-ribose 1-methylphosphonate 5-triphosphate synthase |
| *phnK* | K05781 | C-P lyase subunit, alpha-D-ribose 1-methylphosphonate 5-triphosphate synthase |
| *phnO* | K09994 | C-P lyase subunit, aminoalkylphosphonate N-acetyltransferase |
| *phnM* | K06162 | C-P lyase subunit, alpha-D-ribose 1-methylphosphonate 5-triphosphate diphosphatase |
| *phnJ* | K06163 | C-P lyase subunit, alpha-D-ribose 1-methylphosphonate 5-phosphate C-P lyase |
| *phnI* | K06164 | C-P lyase subunit, alpha-D-ribose 1-methylphosphonate 5-triphosphate synthase |
| *phnH* | K06165 | C-P lyase subunit, alpha-D-ribose 1-methylphosphonate 5-triphosphate synthase |
| *phnG* | K06166 | C-P lyase subunit, alpha-D-ribose 1-methylphosphonate 5-triphosphate synthase |
| *phnP* | K06167 | C-P lyase subunit, phosphoribosyl 1,2-cyclic phosphate phosphodiesterase |
| *phnF* | K02043 | C-P lyase subunit, GntR family transcriptional regulator, phosphonate transport system regulatory protein |
| *ppx* | K01524 | exopolyphosphatase / guanosine-5'-triphosphate,3'-diphosphate pyrophosphatase |
| *ppa* | K01507 | inorganic pyrophosphatase |
| *phnA* | K06193 | phosphonoacetate hydrolase |
| *phnX* | K05306 | phosphonoacetaldehyde hydrolase |
| *phnW* | K03430 | 2-aminoethylphosphonate-pyruvate transaminase |
| *appA* | K01093 | 4-phytase |
| *opd* | K07048 | phosphotriesterase |
| *ugpQ* | K01126 | glycerophosphoryl diester phosphodiesterase |
| *phoA* | K01077 | alkaline phosphatase |
| *phoD* | K01113 | alkaline phosphatase |
| *phoN* | K09474 | acid phosphatase |

**Table S3.** The reference of soil-borne pathongens

|  | Bacteria |  |
| --- | --- | --- |
| Soil-borne pathongens | *Gaeumannomyces graminis* |  |
|  | *Fusarium oxysporum* |  |
|  | *Aphanomyces euteiches* |  |
|  | *Heterodera avenae* |  |
|  | *Heterodera schachtii* |  |
|  | *Meloidogyne spp.* |  |
|  | *Criconemella xenoplax* |  |
|  | *Thielaviopsis basicola* |  |
|  | *Phytophthora cinnamomi* |  |
|  | *Phytophthora infestans* |  |
|  | *Pythium splendens* |  |
|  | *Pythium ultimum* |  |
|  | *Rhizoctonia solani* |  |
|  | *Streptomyces scabies* |  |
|  | *Plasmodiophora brassicae* |  |
|  | *Ralstonia solanacearum* |  |

**Table S4.** The classification, function descriptions and KO number of pathogenic genes referring to KEGG database.

| Classification | genes | KO | Description |
| --- | --- | --- | --- |
| type III secretion systems | *yscC* | K03219 | type III secretion protein C |
|  | *yscD* | K03220 | type III secretion protein D |
|  | *yscF* | K03221 | type III secretion protein F |
|  | *yscJ* | K03222 | type III secretion protein J |
|  | *yscL* | K03223 | type III secretion protein L |
|  | *yscN* | K03224 | ATP synthase in type III secretion protein N |
|  | *yscQ* | K03225 | type III secretion protein Q |
|  | *yscR* | K03226 | type III secretion protein R |
|  | *yscS* | K03227 | type III secretion protein S |
|  | *yscT* | K03228 | type III secretion protein T |
|  | *yscU* | K03229 | type III secretion protein U |
|  | *yscV* | K03230 | type III secretion protein V |
|  | *yscA* | K04048 | type III secretion protein A |
|  | *yscB* | K04049 | type III secretion protein B |
|  | *yscE* | K04050 | type III secretion protein E |
|  | *yscG* | K04051 | type III secretion protein G |
|  | *yscH* | K04052 | type III secretion protein H |
|  | *yscI* | K04053 | type III secretion protein I |
|  | *yscK* | K04054 | type III secretion protein K |
|  | *yscM* | K04055 | type III secretion protein M |
|  | *yscO* | K04056 | type III secretion protein O |
|  | *yscP* | K04057 | type III secretion protein P |
|  | *yscW* | K04058 | type III secretion protein W |
|  | *yscX* | K04059 | type III secretion protein V |
|  | *yscY* | K04060 | type III secretion protein Y |
|  | *HrpB1* | K18373 | type III secretion protein HrpB1 |
|  | *HrpB2* | K18374 | type III secretion protein HrpB2 |
|  | *HrpF* | K18376 | type III secretion protein HrpF |
|  | *HpaA* | K18379 | type III secretion regulatory protein HpaA |
|  | *HpaB* | K18380 | type III secretion control protein HpaB |
|  | *HpaC* | K18381 | type III secretion control protein HpaP |
| type III effector protein | *popP2* | K13478 | type III effector protein PopP2 |
|  | *avrBs3* | K18876 | type III effector protein AvrBs3 |
|  | *xopD* | K18879 | type III effector protein XopD |

**Table S5.** The average relative abundance of biocontrol bacteria

|  | Bacteria | Control | *P. chinense* | *P. scandens* | *M. micrantha* |
| --- | --- | --- | --- | --- | --- |
| Biocontrol Bacteria | *Agrobacterium* | 9.11E-06 | 5.40E-05 | 5.56E-05 | 3.18E-05 |
|  | *Arthrobacter* | 2.46E-05 | 2.99E-05 | 2.65E-05 | 2.34E-05 |
|  | *Azotobacter* | 1.42E-06 | 2.00E-06 | 1.53E-06 | 2.00E-06 |
|  | *Bacillus* | 4.88E-05 | 1.83E-04 | 1.04E-04 | 1.47E-04 |
|  | *Burkholderia* | 2.11E-04 | 1.33E-03 | 2.70E-03 | 1.08E-03 |
|  | *Collimonas* | 3.76E-06 | 4.34E-06 | 3.14E-06 | 2.37E-06 |
|  | *Pantoea* | 0.00E+00 | 9.05E-07 | 3.78E-07 | 2.89E-06 |
|  | *Pseudomonas* | 3.45E-05 | 3.82E-04 | 1.34E-04 | 4.53E-04 |
|  | *Serratia* | 2.60E-07 | 8.82E-07 | 7.73E-07 | 1.36E-06 |
|  | *Stenotrophomonas* | 2.00E-06 | 2.25E-05 | 7.27E-06 | 9.98E-06 |
|  | *Streptomyces* | 8.32E-04 | 2.21E-03 | 1.78E-03 | 1.88E-03 |
|  | *Catenulispora* | 4.78E-04 | 4.70E-04 | 4.70E-04 | 5.59E-04 |
|  | *Candidatus Entotheonella* | 5.69E-05 | 5.53E-05 | 7.55E-05 | 4.41E-04 |

**Table S6.** The classification, function descriptions and KO number of polyketide synthase genes referring to KEGG database.

| Classification | genes | KO | Description |
| --- | --- | --- | --- |
| Type II PKS (aromatic polyketides) | *whiEIII* | K14667 | minimal PKS ketosynthase (KS/KS alpha) |
|  | *whiEIV* | K14668 | minimal PKS chain-length factor (CLF/KS beta) |
|  | *whiEIV* | K14669 | minimal PKS acyl carrier protein |
|  | *whiEIVI* | K14670 | aromatase |
|  | *whiEIVII* | K14671 | cyclase |
|  | *actI1* | K05551 | minimal PKS ketosynthase (KS/KS alpha) |
|  | *actI2* | K05552 | minimal PKS chain-length factor (CLF/KS beta) |
|  | *actI3* | K05553 | minimal PKS acyl carrier protein |
|  | *actIII* | K12420 | ketoreductase |
|  | *actVII* | K05554 | aromatase |
| Type III PKS | *phlD* | K15431 | phloroglucinol synthase |
|  | *rppA* | K19580 | 1,3,6,8-tetrahydroxynaphthalene synthase |
|  | *gcs* | K16232 | germicidin synthase |
|  | *pks18* | K16233 | alpha-pyrone synthase |

Supplementary Figure 1. The relative abundance of *gcd* gene in the rhizosphere of three plants and control soil. Error bars indicate average value ± s.e.m. of indicated replicates. The pairwise comparisons of rhizosphere in each plant and control soil were used by the Kruskal Wallis test with Dunn's multiple comparison test (*P<0.05 and **P<0.01).
